# The Æsthetics of Suicide

**Published:** 1859-10-01

**Authors:** 


### Art. VII.—THE ÆSTHETICS OF SUICIDE.

"The suicide does not undergo death because it is honourable, but in order to avoid evil."—*Aristotle.*

"Do you know, [said Socrates, that all except philosophers] consider death among the great evils?"

"They do indeed," [Simmias] answered.

"Then do the brave amongst them endure death, when they do endure it, through dread of greater evils?"

"It is so."

"All men, therefore, except philosophers, are brave through being afraid and fear; though it is absurd that any one should be brave through fear and cowardice."—*Plato.*

In the north room of the Royal Academy, at the last Exhibition, there was hung a painting, the subject of which was somewhat singular. It represented a garret, within which was depicted, sitting at the edge of a truckle bed, a young man whose countenance had a scared aspect. At his feet, upon the floor, sat a woman, her form huddled together, her head resting upon his knees, and her face hid by her arms. Nigh at hand, in front of an overturned stove, was a little heap of fiercely burning charcoal, and on a table, in the corner of the room, might be distinguished the butt-end of a pistol. Through a curtainless window could be seen the tops of green trees, and a patch of blue sky; while the pale light of early morning, or of the closing eventide, and the fiery glow of the burning charcoal, lit the scene.

We have described the picture from memory, and may perhaps have erred in some of the slighter details, but the chief characteristics were such as we have given.

The artist had done his work featly, and it did not need a second glance to see that he had fixed upon the canvas, with no contemptible skill, a too common phase of every-day-life suicide. But what recent or sometime past instance of double suicide so far appealed to our sympathies, or what description of such an event in literature stood so markedly prominent, from the excelling power of the writer, that the pencil should add a halo to the ghastly incident, or to the writer's pen? We turned to the catalogue, but appended to the number of the painting there was simply this sentence—

"*The fumes of charcoal.*"

On the walls of the same Academy, a few years before, Wallis's wonderful painting of Chatterton's self-murder had hung. This suicide, however,—the wretched end of one of the most conspicuous examples of misdirected genius that the world ever saw, claims a place in history. Of the limner's representations of everyday suicide, we know M. Decamp's horrible but nervous drawing. A young man, wasted by suffering and half naked, is extended upon a wretched bed, in an equally wretched attic. A blanket, the sole clothing, envelopes the body. The head has fallen backwards, and the long, trailing, entangled hair is dabbled with blood. One hand reposes on the breast, the other rests flaccidly upon the floor. Near the bed lies a still smoking pistol, while against the wall lean an easel and a palette, upon which the colours are still moist. On a rough-hewn plank above the easel are arranged a few books, and alongside them stand a plaster statuette, and a death's-head. This painting is simply termed *The Suicide*, and in nowise is the terrible story which it tells, or the terrible lesson which it conveys, mitigated or distorted. We know also Cruikshank's too truthful drawing, the last of the series named *The Drunkard's Children*, a sequel to *The Bottle*. Who has not shuddered when he has gazed upon the agonized figure, which, with the hands convulsively clasped upon the eyes, has sprung from the parapet of the bridge? How vividly the mind pictures to itself the sullen wash of the river against the piers; the dark glassy surface of the water in the huge black shadow of the masonry; the golden gleam of the moonshine on the distant ripples. How involuntarily we shiver at the thoughts of the chilly, damp air which hovers over the stream, and sicken at the awful sense of solitariness which is apt to steal over one when, standing on one of the bridges, he is hemmed in by the midnight sounds of the city. Alas! for the friendless who at such an hour and on such a spot may listen to them!* Then the sharp, painful recoil of the feelings, as the slight, scarcely heard splash strikes the ear from below, and the eager gaze with which we peer into the gulf, and mark the two or three pale, fleeting gleams of silvery light which crest the diminutive waves, tossed up by the cloven water. "God help the poor unfortunate!" we exclaim; rather should we cry, "*God help us*"—to read aright, and to act aright after having read, the legend written beneath the picture—"*The maniac father and the convict brother are gone! The Poor Girl, homeless, friendless, deserted, destitute, and gin-mad, commits self-murder!*"

"Alas! for the rarity  
Of Christian charity  
Under the sun!  
Oh! it was pitiful!  
Near a whole city full,  
Home she had none."

* "When I first saw the river as I passed over King's College Bridge," said Robert Hall, speaking of the Cam, to Dr. Olinthus Gregory, "I could not help exclaiming, Why, the stream is standing still to see people drown themselves! and that, I am sorry to say, is a permanent feeling with me." How many, doubtless, are affected by a very similar feeling on looking at night upon the Thames, where

We know also that little sketch of Thackeray's, which illustrates "*A Gambler's Death.*" It is but a roughly executed drawing, but it is *taken from nature*, and set in the tale to which it belongs, is of surpassing interest. (See the *Paris Sketch-Book.*)

Decamp's, Cruikshank's, and Thackeray's drawings tell the rigid, ghastly truth of everyday suicide; and the drawings of the two latter men convey a lesson not easily overlooked or forgotten, and free from a certain terrible fascination, which rivets the attention to the painting of the former man, even from its very truthfulness. But the "*Fumes of Charcoal*" is a picture which, judging from its title and execution, aims at depicting suicide from an æsthetical point of view. The horrible character of the act is partly overshadowed by a certain unhealthy sentiment, and the painting appeals to a morbid sympathy, rather than to a sound feeling of abhorrence tempered with an active and well-directed pity.

We may be wrong in our estimate of the artist's work, and we hope we are. It may, moreover, be simply an illustration of peculiar notions on his part of the functions of the painter's art; but we cannot help regarding this painting as one of several indications, which seem to point to a growing sympathy towards the act of suicide in this country.

Our coroners' juries have become so sensitive of the fame of suicides, that (so far as can be judged from newspaper reports*) the rule is to pronounce a verdict of temporary insanity in cases of self-murder. This legalized apology for suicide is not only too often an evasion both of the law and the gospel, but it tends to throw discredit upon the doctrine of temporary insanity, and what is even still more important, to convert the act of suicide into an object of legitimate pity.

We have no special literature of suicide in England at the present time; but our Gallic neighbours supply us in this respect superabundantly. With them suicide holds a very similar position in popular writings, and is invested with the same kind of sentiment as "broken-hearts" and consumption with us. We meet with self-murder at every turn in some of the most popular and widely-spread forms of French literature, and the act is clad with so many charms of a highly æsthetical character, and altogether takes so respectable a position among the legitimate causes of death, that one recoils in fear from the insidious doctrines (absurd though they may be) implied or taught. Witness the *Mémoires d'un Suicide recueillis et publiés par Maxime du Camp*, (Paris, it winds within the metropolis,—a feeling often increased if not originally prompted by the sad associations connected with the bridges. It is to be feared that many who have been foiled at every turn in life's struggle, have become fascinated with the terrible idea and yielded to it.

* Cannot Mr. Samuel Redgrave help us to some more definite and satisfactory information upon this subject?

1855,) and the more recent work, *Les Suicides Illustres : biographie des personnages remarquables de tous les Pays qui ont péri volontairement depuis le commencement du Monde jusqu'à nos jours, par F. Dabadie* (Première Série. Paris, 1859.)

Charles Nodier had conceived the notion of writing the biography of noted suicides, and reading to us the "solemn philosophical lesson" which is to be derived from the string of renowned artists, poets, inventors, legislators, heroes, conquerors, kings, queens, emperors, priests, and lovers who have murdered themselves. "It is singular," remarks M. Sartorius, in a prefatory notice to M. Dabadie's work, "that for the last thirty years we have been swamped with celebrated brigands, celebrated kings, celebrated wives, celebrated children, celebrated animals, &c., but no one has recounted the tale of celebrated suicides. Thanks, however, to M. Dabadie, this much to be regretted hiatus in the French book-trade has been (by our advice) filled up."

We have the book, but the solemn philosophical lesson, and the biographical research, which would have legitimized its place in literature, are wanting. "*Les Suicides Illustres*" appeals to the *dilettanti* in suicide. In his Introduction to the work, M. Dabadie, after having touched in a slip-shod fashion upon several opinions respecting suicide, writes :—

"Morality—we speak of social not of religious morality [happy distinction !] which has nothing to do here—morality, we say, which is elevated above the law by its origin, its function, its end, and which moves in a more extended sphere, justly disapproves of certain suicides. For example, every married man has contracted a sacred engagement; he owes help and protection to the woman he has espoused, as also to his children. This engagement having been freely entered into, one thing only can dissolve it—the radical and definitive impossibility of fulfilling it. Thus the father of a family who is or can be useful to it, is blameable if he disembarrass himself of that life which does not belong to him.

"To recall this incontestible principle is to demonstrate that man has not *always* the right to kill himself. But universal opinion would be wounded—and we are tempted to add morality (!)—if it were sustained that he has *never* the right. In truth, in the eyes of opinion, there are suicides which are not only excusable but even praiseworthy. Such is the suicide of the commandant of a fortification or of a ship which he blows up rather than render it to an enemy. Orators, poets, and celebrated historians, as well as heroes, soldiers and sailors, who have had the bravery to accomplish this resolution, are admired by the people, and the Church dare not refuse to pray for the health of their souls. More than once it has happened that suicide has been the brevet of glory. Witness those Greeks and Romans who fell so nobly that *death was proud to take them*, according to the magnificent expression of the English poet; and without going so far back, the young officer of our navy (Bisson), who immortalized himself in the waters of the Archipelago, under the Restoration.

"As to the vulgar suicides, it appears to us better to pity than to blame them. Rich or poor, old or young, ill or well, man is bound to existence by so many ties—without noticing the bond of habit—that he must have suffered cruelly before conceiving the idea of destroying himself, especially before realizing it. . . . . ." (pp. xxv—xxvii.)

Let it not be supposed that notions such as these are maintained by obscure writers solely. We may mark an approximation to them in a recent expression of opinion by one who has an enviable position among physicians, and whose scientific writings on suicide have a world-wide reputation—Brierre de Boismont. In the course of an inquiry into the suicidal or accidental nature of an injury which had occasioned the death of a gentleman in Paris in September, 1858, M. Pinard, the substitute of the procureur-impériale, said :—

"We are not of those too-austere legislators who without pity for the dead would gibbet the bodies of suicides, and drag them through the streets upon a hurdle.* We live, on the contrary, in the midst of an enfeebled society which beholds with indifference the multiplication of suicide, and which regards it more with pity than with anger. Does society look upon self-murder as a good or an evil? To listen to certain doctrines and to witness the ravages of this evil extending into all classes of society, we should say that it has doubts in this respect, and that it forgave all those who had recourse to it. Neither need we wonder at these doubts when we meet with poets who say to distempered souls, death is a sleep; rest ye and break the vase if the liquor is too bitter: when we encounter more hardy minds who proclaim to all that death is a right and the disinherited may quit a world which has abandoned them. Against this double cry of feebleness and pride it is necessary that we should maintain the old principles that have been taxed as common-place (as if common-places were not eternal truths), that suicide which arises from madness is a calamity, that when it is committed by a sane person it is a crime.

"Is not suicide a protest against the life to come, a protest against the immortal principle we carry in us, a protest against the social duties which we have given rise to and which we ought to fulfil to the end? Then ought every flourishing society to guard against this disease of eternal faiths. Then ought magistrates always to regard suicide as a disgrace, a crime to be engraved on a tomb, a dishonour bequeathed to a family."

Upon these opinions M. B. de Boismont remarks :—

"We are keenly affected by these noble and generous words, but do they not admit of any exception?

* "1598, February 20.—The 20 day of Februar, Thomas Dobie drownit himself in the Quarrel holes, besyde the Abbey, and upon the morne he was harlit throw the towne backward, and thereafter hangit on the gallows."—*Robert Birrel's Diary—Notes and Queries*, vol. v., p. 272.

"Philip Strozzi had fallen into the hands of his most cruel enemy, Come de Medicis, whom he had wished to overturn. He was one of a body of conspirators, of whom he possessed the secrets. If he spoke, their heads would roll upon the scaffold, their property would be confiscated, their families proscribed and reduced to indigence, and his name and himself would be dishonoured. If he had but to meet an ordinary death his silence would not be shaken, but torture might triumph over his courage, as it had triumphed over that of the unfortunate Julian Gondi and many others, and cause him to forswear himself. He would not brave a like peril. Filled with the learning of the ancients, whose works had been recently disinterred, after many ages of darkness, and had electrified Italian imaginations, he descended to the tomb, invoking the name of Cato, and of those virtuous men who had likewise killed themselves. If Strozzi be criminal, truly his crime is of a nature every way peculiar, because his memory does not lack the sympathies of many men, and his memory will always be respected.

"In the midst of the agitations which disturb the world, perhaps there would be fewer villanies, and more great actions, if those who are called to play a part upon the political scene took the resolution to die rather than to abandon the triumph of their ideas, or preferred honour to life. There are epochs, says M. S. De Sacy, when to die with readiness is a noble science; and if Christianity, from a more elevated point of view, condemns absolutely suicide, after the courage of maintaining life in obedience to God, it must be admitted that there is no greater courage than that of quitting it voluntarily in order to avoid being sullied by a baseness."—*Recherches Médico-Légales sur le Suicide à l'occasion d'un cas douteux de mort accidentelle ou violente. Par A. B. de Boismont. Annales d'Hygiène Publique. July*, 1859.

M. B. de Boismont's reasoning would leave a tolerably wide path open and an ample verge, capable of unlimited enlargement, for suicide. For who is to lay down those rules which would enable us to determine with precision the circumstances when suicide becomes not merely justifiable but even praiseworthy? Honour and the world's-opinion are not synonyms of virtue as the world goes, and to take them as guides would leave us in precisely the same predicament that the world has been in with regard to suicide ever since it began to play a conspicuous part in history.

If M. Boismont's *in extremis* doctrine of political conduct were adopted, it is evident that it would not be the *ideas* contended for, but the success or not of those ideas which must govern the act of self-murder, for the doctrine is applicable to every shade, every variety of belief entertained by politicians. Think for a moment of Louis Napoleon struggling against the evils of penury, of expatriation, nay, of seemingly hopeless exile in a back street of London; his most cherished notions crushed; his greatest efforts not merely unsuccessful, but a mark of ridicule. Picture to yourself this man discovered one morning, amidst all the bustle and hurry of the huge city, stretched upon his bed, his head shattered, and the instrument of the foul deed within the grasp of the stiffened fingers; picture the stolid jury; the remarks of contemptible pity; the execrations of creditors; and the final interment in some obscure spot of one of the many desolate burial-grounds, or of the crowded cemeteries of London. Yet in what instance could M. Boismont's aspiration concerning self-murder have been more justified? But think of Louis Napoleon, Emperor of the French, and making for himself a colossal name in history, and reflect on the horrible absurdity of suicide as a last political resort. This is no question of Buonapartism or not; for the lesson belongs to every creed of political faith, but it is of the most value to those creeds which are for the nonce depressed, nay apparently hopeless.

Again, sympathy with the motives which may have led to self-murder is one thing; to hold them up as lights for our guidance is another and very different thing. Philip Strozzi dying on the rack, silent amidst his torments, or throwing a noble scorn at his executioners; Philip Strozzi emulating the courage of many Christian martyrs and warriors, and not the bastard courage of a Cato, would have as far excelled the Philip Strozzi dying by his own hand in a dungeon, as Lucifer the angel excelled Lucifer the fallen one. But, alas! for the ill-example to his supporters—alas! for any cause whose adherents are taught so ready a way to avoid difficulties; his physical courage failed, and Florence lost the example of a martyr and got the old-fashioned one of a mere conspicuous man. We pity Strozzi, but we should no more hold him up as an example to be followed, than the Red Indian would hold out to his son as an example the man who, to avoid torture when a captive, had destroyed himself, or who had suffered a groan to escape him under the torture.

It is recorded that when Charles V. was told of Strozzi's death, and the fashion of it, he remarked, *smiling*, "may all my enemies perish thus." It may be surmised that the Emperor shrewdly suspected that Strozzi dying by his own hand would have a less exciting effect upon the Florentines than Strozzi dying by the executioner. The Emperor's opinion of Strozzi and his co-workers, as expressed to Antonio Doria, is not to be overlooked when the question of the patriot's death is noted for admiration. "You little understand these men," said Charles V.; "they do not wish the liberty of their country, but their own greatness; for if we were to remove the duke, they themselves would become lords of Florence, in spite of the citizens, who really love the liberty of the city, but who could not resist the influence, and wealth, and power of these ambitious leaders."

The most transcendental of æsthetical views concerning suicide is that of Elias Regnault (*Nouvelles Réflexions sur le Suicide*), quoted by M. Dabadie. M. Regnault writes :—

"Suicide is the last term, the highest expression of man's liberty. It is the most energetic protest of the superiority of his nature. Why have not animals ever conceived suicide? Because their nature is every-way passive. They have not the choice and the preference. Man, on the contrary, eminently active and free, has been able to push his activity even to the destruction of himself."

The thought is borrowed from Pliny, but it is a pity that it should have been truncated by Regnault. Here is the missing fragment :—"Indeed," says the pagan writer, "this constitutes the great comfort in this imperfect state of man, that even the Deity cannot do everything. For he cannot procure death for himself, even if he wished it, which, so numerous are the evils of life, has been granted to man as our chief good."—(*Nat. Hist.* B. II. c. V.)

But Regnault and his co-thinkers have it that suicide is "la manifestation la plus éclatante de la personnalité humaine," only when the act is essentially voluntary. Suicide under the influence of anger or mental alienation is exempted from the category. And really if, with these reservations, it were accepted that suicide is the highest expression of man's liberty, it would simply lead to the conclusion that the act of self-murder involves the highest degree of his responsibility, social, moral, or religious.

A more recent apology for suicide than Regnault's, and one much more novel and curious, is that of M. Bourdin. He holds that suicide is suicide only under certain circumstances. He writes :—

"Sacred and profane history furnish us with many examples of men who have exposed themselves seriously and voluntaril to death, with-y out having nevertheless committed suicide. For example, Samson, become blind, buries himself beneath the ruins of a temple which he has overturned. Eleazar suffers himself to be crushed to death by the falling of an elephant which he has killed. Epaminondas, after having asked if his shield was safe, wishes the javelin to be torn from him, although its removal will cause death. Curtius devotes himself to the gods, and casts himself into a gulf to save his country. Regulus returns to Carthage, loving better to meet death than to violate his sworn faith. Christian history is filled with edifying examples of holy women who have preferred to expose their life than undergo a shame (*potius mori quam fœdari*). Saint Domnine and her two daughters, Saints Berenice and Prosdoce, drowned themselves in order to save their chastity; Saint Pelagie and her mother threw themselves from a roof to evade the violence of the governor of Antioch.—(Saint Ambrose, *De Virginibus*, lib. iii.) Saint Ignatius, bishop, wished that the faithful at Rome should not sue for his pardon: *Voluntarius moriar, inquit, quia mihi utile est mori.* It would be easy to cite a great number of sacrifices as generous, inspired by faith, by political beliefs, or even by tender but exalted sentiments, such as love, friendship, &c. In these different acts are not found the characteristics of suicide; because to expose one's self to death, to place one's-self even in circumstances which render death inevitable, is not to wish to kill one's-self—is not to act with a formal and exclusive intention of killing one's-self.

This delectable piece of reasoning is somewhat akin to a casuistical opinion of Luther's. He was told of a young girl who, to avoid violence offered to her by a nobleman, had cast herself out of a window and was killed. The question was asked, was she responsible for her death? Luther said, "No: she felt that this step formed her only chance of safety, it being not her life she sought to save, but her chastity."—(Luther's *Table Talk.*)

"If," to continue M. Bourdin's remarks, "suicide does not exist in the conditions that I have named, all the more does it not exist in regard to those tender but passionate souls, who, feeling the emptiness and nothingness of all around them, ardently lay claim to another country. Still less does it exist in the instances of those members of the National Convention, who, as it is said, *have committed suicide to maintain their honour.* This last distinction is not as vain as it may appear to be at the first sight, because the confusion that it destroys has been made by able thinkers who have not sufficiently studied the matter.

"This preliminary explanation was necessary in order to destroy every species of equivocation and to define exactly the limits within which suicide exists; it was necessary also, in order to eliminate from the pathological classifications of suicide those facts which do not belong to them."—(*Du Suicide considéré comme Maladie.* Paris, 1849.—p. 9.)

When, therefore, as the result of his researches and of "simple inductive ratiocination," M. Bourdin writes, "I say that suicide is always a disease and always an act of mental alienation; I say, consequently, that it does not merit either praise or blame," (*Op. Cit.* p. 9); we know that he is not using the term suicide in its ordinary sense.

M. Bourdin's conclusion that suicide, in his restricted sense of the word, merits neither praise or blame, would, however, seem to be the right deduction to attach to a certain *quasi*-scientific theory of suicide, in the most extended sense of that term, which has been declared by Mr. Buckle in his *History of Civilization in England.* He asserts that all the evidence we possess points "to one great conclusion, and can leave no doubt on our minds that suicide is merely the product of the general condition of society, and that the individual felon only carries into effect what is a necessary consequence of preceding circumstances."

The reasons adduced for so remarkable a conclusion deserve to be gravely considered. Mr. Buckle writes:—

"Among public and registered crimes, there is none which seems so completely dependent on the individual as suicide. Attempts to murder or to rob may be, and constantly are, successfully resisted; baffled sometimes by the party attacked, sometimes by the officers of justice. But an attempt to commit suicide is much less liable to interruption. The man who is determined to kill himself is not prevented at the last moment by the struggles of an enemy; and as he can easily guard against the interference of the civil power, his act becomes, as it were, isolated; it is cut off from foreign disturbances, and seems more clearly the product of his own volition than any other offence could possibly be. We may also add, that, unlike crimes in general, it is rarely caused by the instigation of confederates; so that men, not being goaded into it by their companions, are uninfluenced by one great class of external associations, which might hamper what is termed the freedom of their will. It may therefore very naturally be thought impracticable to refer suicide to general principles, or to detect anything like regularity in an offence which is so eccentric, so solitary, so impossible to control by legislation, and which the most vigilant police can do nothing to diminish. There is also another obstacle that impedes our view: this is, that even the best evidence respecting suicide must always be very imperfect. In cases of drowning, for example, deaths are liable to be returned as suicides which are accidental; while, on the other hand, some are called accidental which are voluntary. Thus it is that self-murder seems to be not only capricious and uncontrollable, but also very obscure in regard to proof; so that on all these grounds it might be reasonable to despair of ever tracing it to those general causes by which it is produced."

Are the circumstances and the motives which lead to or determine the act of suicide so exceptional as to present no aspect, even at a slight glance, of regularity of recurrence? Have the assumed remote and proximate causes of the deed been of so erratic a character, and so seemingly irregular in their manifestation, as to exhibit no indications of uniformity of action? Have the many physical and psychical troubles which have impelled man to destroy himself played so unimportant a part in the history of society and of races, manifested such marked characteristics of the incidental and not of the general, that any one of the results to which they have given rise should be expected to present a "capricious" stamp? Is self-murder "so rarely caused by the instigation of confederates:" is this the lesson taught by the history of suicide among the Greeks and the Romans of old, the Japanese, the Hindoos, and the Parisians of our own days? Do experience and history show that the motives which affect the volition, which bring the mind into the state of pleasing to do a thing or not,* are so different in different people; do they show that the operations of the emotions and of the thoughts, as well as the action of the motives which influence them are so eccentric, that "what is termed the freedom of the will" is alike and manifestly eccentric? To each and all of these interrogatories all ordinary individuals, we have little doubt, would unhesitatingly answer, No! Why, it seems to us that all *à priori* reasoning hitherto has led to the very reverse of Mr. Buckle's assertion that it might "very naturally be thought impracticable to refer suicide to general principles, or to detect anything like regularity in an offence which is so eccentric." That the recurrence of suicide was governed by definite laws is a belief as clearly implied in the writings of the ancients upon the act, as that the conviction in the existence of those laws has been a principal incentive to frequent research concerning suicide in all its aspects among the moderns.

As to the *impossibility* of controlling self-murder by legislation and a vigilant police, that is a question of fact which Mr. Buckle deals with as if it were a mere matter of opinion, for he contents himself with the bare assertion at some length, and a reference or two which may, perhaps, be quoted legitimately by those who hold the opinion of the *inutility* of the present system of legislation on suicide, but can afford only slight or very problematical grounds for the belief in the impossibility of controlling suicide by any legislation. We shall have to examine this subject at a greater or less length in a subsequent portion of this article, consequently, we shall simply make here the additional remark, that the fullest and most careful account that we are acquainted with of the history and legislation of suicide among different nations, that of Lisle's (*Du Suicide. Paris*, 1856,) shows not only that there is no sufficient foundation for the opinion that legislation, at all times and under all circumstances, is inoperative in checking suicide, or that the belief of lawgivers that by their enactments they can diminish suicide, is, as Mr. Buckle asserts, "folly," (*Note*, p. 24,) but also that there is good ground for hope that well-considered legislation would prove beneficial in checking or controlling the evil.

Mr. Buckle's preliminary propositions are in the main mere assumptions. But to continue his argument:—

* *Bailey. Letters on the Philosophy of the Human Mind. 2nd Series.—p.* 173.

"These being the peculiarities of this singular crime, it is surely an astonishing fact, that all the evidence we possess respecting it points to one great conclusion, and can leave no doubt in our minds that suicide is merely the product of the general condition of society, and that the individual felon only carries into effect what is a necessary consequence of preceding circumstances. In a given state of society, a certain number of persons must put an end to their own life. This is the general law; and the special question as to who shall commit the crime, depends, of course, upon special laws, which, however, in their total action, must obey the large social law to which they are all subordinate. And the power of the larger law is so irresistible, that neither the love of life nor the fear of another world can avail anything towards even checking its operation."

Now, notwithstanding that Mr. Buckle states that "all the evidence we possess" points to this conclusion, he refers only to four sources of evidence, Dufau's *Traité de Statistique,* Winslow's *Anatomy of Suicide,* Quetelet's *Statistique Morale,* and certain tables in the *Assurance Magazine.* Certainly one cannot help admiring the hardihood of fixing so magnificent a conclusion on the confessedly and necessarily slender data contained in these works. Why we assume that Mr. Buckle's references constitute at least the head and front of his "all the evidence we possess" will be seen presently. As indicating the value of these references in relation to Mr. Buckle's conclusion, we may remark that Quetelet seems to constitute his chief statistical authority, and he is spoken of by him as "confessedly the first statistician in Europe," a sentiment which one might suppose would have at least induced Mr. Buckle to respect his opinions. Now Quetelet has expressly defended his researches from the conclusions which Mr. Buckle is desirous of attaching to them. Quetelet has written—

"That which precedes shows us that man, in general, proceeds with the greatest regularity in all his actions. Whether he marries, begets, kills himself, robs, or murders, he invariablyseems to act under the influence of definite causes independent of his free-will.

"We must carefully guard ourselves here, nevertheless, from concluding that this constancy is the result of a desolating fatalism. FOR OURSELVES, WE SEE IN IT BUT THE PROOF OF THE PERMANENCE OF THE MORAL CIRCUMSTANCES WHICH GIVE RISE TO SUICIDES, DURING THE PERIOD WHICH OUR OBSERVATIONS EMBRACE."*

* "Tout ce qui précède nous montre que l'homme, en général, procède avec la plus grande régularité dans toutes ses actions. Qu'il se marie, qu'il se reproduise, ou qu'il se tue, qu'il attente à la propriété ou à la vie de son semblable, toujours il semble agir sous l'influence de causes déterminées et placées en dehors de son libre arbitre.

"Nous nous garderons bien cependant de conclure de là, que cette constance est le resultat d'un fatalisme désolant. Nous n'y voyons, pour nous, que la preuve de

Again, Mr. Buckle's assertion that "suicide is merely the product of the general condition of society, and that the individual felon only carries into effect what is a *necessary* consequence of preceding circumstances," is much the same kind of proposition as if it were said that the quotient determined the value of the different figures and the method of working of a sum. "In a given sum," to adopt Mr. Buckle's phraseology, "certain results must follow. This is the general law; and the special question as to what position each figure shall take in the sum depends, of course, upon special laws; which, however, in their total action, must obey the large arithmetical law to which they are all subordinate. And the power of the larger law is so irresistible, that neither the vexations of multiplication, nor the still greater troubles of division, nor the perplexities of rule of three, nor the maddening irritations of vulgar fractions,* can avail anything towards checking its operation." Thus the different psychical and physical elements, which are usually supposed to concur in forming the general result commonly spoken of as the state or condition of society, are to be regarded as having their value defined, or regulated, or governed by the results to which they have given rise; or the psychical elements are to be looked upon as not being concurrent causes with the physical in bringing about the state of the society, the former elements being products of the latter, which, in some unexplained manner, constitute or engender the general state spoken of; or the said state of society is a something *per se*—an active entity, or anything, or nothing, as the case may be. The first supposition strikes one as the meaning of Mr. Buckle's proposition at the first glance; the second is necessary to explain certain peculiarities of his explanation of that proposition; the third will be found generally useful in reading the introductory chapters of his work, and the continuation of his argument, which proceeds thus:—

"The causes of this remarkable regularity I shall hereafter examine; but the existence of the regularity is familiar to whoever is conversant with moral statistics. In the different countries from which we have returns we find year by year the same proportion of persons putting an end to their own éxistence; so that, after making an allowance for the impossibility of collecting complete evidence, we are able to predict, la permanence des circonstances morales qui font naître les suicides, pendant la période qu'embrassait nos observations."—(*Quetelet. Du Système Social et des Lois qui le régissent. Paris.* 1848.—p. 327.)

* Multiplication is vexation,
Division's twice as bad,
Rule of Three it puzzles me,
And Fractions make me mad.—*School Song.*

within a very small limit of error, the number of voluntary deaths for each ensuing period; supposing, of course, that the social circumstances do not undergo any marked change."

In fact, suicide is subject to the ordinary laws of causation. Then what are the "social circumstances" spoken of which are liable to variation? They are not of a moral character, because Mr. Buckle teaches "that the moral actions of men are the product not of their volition but of their antecedents" (p. 29); and that "suicide is merely the product of the general condition of society," and of "preceding circumstances." It is evident, therefore, that the term "social circumstances" is not used here by Mr. Buckle in the sense in which it is ordinarily used, and that it is equivalent to the terms "general condition of" and "state of society," "antecedents," and "preceding circumstances," as used by him; and that the two former phrases as well as the two latter are used in some peculiar sense. This sense seems to be capable of no other explanation than a something *per se*—an active entity; and Mr. Buckle, in endeavouring to escape from a metaphysical Scylla has apparently plunged into a profounder Charybdis.

But how do Mr. Buckle's assertions, that "in the different countries from which we have returns, we find year by year the *same* proportion putting an end to their own existence," and "that we are able to predict, within a small limit of error, the number of voluntary deaths for each ensuing period; supposing, of course, that the social circumstances do not undergo any *marked* change," tally with the facts and his authorities? We have not, unfortunately, Quetelet's *Statistique Morale* by us, but there are certain remarks in his work on Man, freely referred to by Mr. Buckle, which have a direct bearing on this question. In that work, Quetelet bases his observations on the annual variations of suicides, on *five* years' records of suicides in France, *ten* years in the department of the Seine, and *seven* years in the Canton of Geneva; and he states, "we recognise in all the preceding figures a frightful concordance between the results of the different consecutive years. This regularity in an act which appears so intimately bound to the volition of man, is manifested more strikingly (as will be presently shown) in all that appertains to crime. Nevertheless, society may be modified in a country, and bring about changes in that which offers, at the first, a remarkable constancy *for a short period* (qui offrait d'abord une constance remarquable pour une période de temps peu étendue). According to Dr. Casper, 62 suicides were committed in Berlin from 1788 to 1797, 128 from 1797 to 1808, and 546 from 1813 to 1822." (*Sur L'Homme*, L. II., c. ii. s. ii.) Quetelet, indeed, tells us in effect, that the regularity in the recurrence of suicide, although true for the "période de temps peu étendue," to which his data referred, cannot be assumed to be true of any other period, unless there be other and more extended observations, because such a conclusion would be inconsistent with Dr. Casper's researches, and unwarranted by the very brief character of his own researches. And such, if our memory serves us right, is the carefully guarded character of all M. Quetelet's researches concerning the annual recurrence of suicide, a care rendered necessary from the comparatively limited character of the statistics with which he had to deal.

Let us here glance for a moment at the French statistics of suicide, and see how they bear upon Mr. Buckle's assertion of the yearly recurrence of suicides in the same proportion, except when marked changes in society occur. In France the number of suicides in proportion to population, was in 1836, 1 in 14,207. From this year there was a progressive increase in the number of suicides year by year, with six exceptions (1841–44–45–46–48–49), until 1852, when the number had increased to 1 in 9340! (*Lisle, Du Suicide*, p. 22.) This is scarcely compatible with Mr. Buckle's assertion.

But again, Dufau's statistics of suicide refer to France, and are confined to the *ten* years 1827–37. In the former year the proportion of suicides to population was, according to him, 1 in 20,660; in the latter, 1 in 14,338 (13,683, *Lisle*), consequently the French statistics of suicide show a steady increase from 1827 to 1852, and this, with little variation, from year to year. But M. Dufau's returns, considered alone, from the limited period of time to which they refer, although very suggestive, afford but a slight foundation for any general conclusions, and so conscious is he of this truth, that he carefully avoids doing anything else than setting forth the facts told by his figures, and when he points out an interesting relationship which seemingly exists between the prevalence of suicide and the mean age of the population of a district, he immediately adds that "we state this simply as a conjecture. The *investigation relative to suicide has but commenced*," *&c.* (*Op. cit.* p. 302.)

If we now take Mr. Buckle's third authority. Dr. Winslow's work, we shall find in the chapter on the statistics of suicide, first, an account of the number of suicides committed in London for a century and a half, the bearing of which on Mr. Buckle's notions we shall have to refer to presently. Then Dr. Winslow quotes the interesting report of a committee of the Statistical Society, on suicides in Westminster, which is preceded by the very proper remark, that "The committee deems it right to premise that caution must be used in drawing too general inferences from these statements, on account of the comparatively small number of cases to which they refer." Next follows an outline of M. Guerry's researches, the value of which will be best shown by his own words,—"These first attempts rarely lead then to an immediate application; they destroy error rather than establish truth, and their utility consists *less in giving rise to theories than in developing the spirit of criticism and research*" (et leur utilité consiste moins à élever des théories qu'à répandre l'esprit de doute et d'examen). (*Essai sur la Statistique Morale de la France*, p. 69.) Lastly follows an account of M. Prevost's researches on suicide in the Canton of Geneva, for the ten years 1825–34, which, as they show (putting aside the short period of observation) an annual mean of 13, a minimum of 6 (1825–1826), a maximum of 24, and a difference of 18, with a population increasing at the rate of about 500 a year, can hardly be supposed to aid Mr. Buckle's ideas much.* Of the value of the statistics of suicide for the metropolis, we shall take Mr. Buckle's own opinion. After referring (*note*, p. 27) to Mr. Jopling's paper on the subject, in the *Assurance Magazine*, Mr. Buckle adds, "These are the only complete consecutive returns of London suicides yet published [they extend over five years], those issued by the police being imperfect."

Now the foregoing is the character of the references of Mr. Buckle concerning the statistics of suicide, yet he precedes his remarks on suicide and murder with the following sentence respecting the statistical evidence on crimes. "This evidence has gone on accumulating until it now forms of itself a large body of literature, containing, with the commentaries connected with it, an immense array of facts, so carefully compiled, and so well and clearly digested, that more may be learned from it respecting the moral nature of man, than can be gathered from all the accumulated

* The following is an approximative calculation of the proportion of suicides to population in the Canton of Geneva, from 1825–34, according to the data given by M. Prevost. Population, 1822, 51,113; 1834, 56,655:—

| *Suicides in* 10,000 *Population.* | | *Suicides in* 10,000 *Population.* | |
|---|---|---|---|
| 1825 | 1.1 | 1830 | 2·9 |
| 1826 | 1·1 | 1831 | 3·2 |
| 1827 | 1·6 | 1832 | 2·2 |
| 1828 | 2·0 | 1833 | 4·1 |
| 1829 | 2·0 | 1834 | 2·8 |

In the thirteen years 1838-47, 1853-55, the annual number of suicides in the Canton of Geneva ranged from 11 to 20, the average being 15·6. These figures exhibit a much less degree of variation than those for 1825-34, and show clearly the necessity for a long period of observations before any very absolute rules can be laid down respecting the annual recurrence of suicides in a country.—(See Dr. Marc d'Espina's *Essai Analytique et Critique de Statistique Mortuaire Comparé.*—(p. 93, *et seq.*)

experience of ages." (!) It is certain that the statistics here spoken of are not those made use of by Mr. Buckle in his examination of the question of suicide.

But to continue Mr. Buckle's argument, lest an iota of it should be lost:—

"Even in London, notwithstanding the vicissitudes incidental to the largest and most luxurious capital in the world, we find a regularity greater than could be expected by the most sanguine believer in social laws; since political excitement, and the misery produced by the dearness of food, are all causes of suicide, and are all constantly varying. Nevertheless, in this vast metropolis, about 240 persons every year make away with themselves; the annual suicides oscillating, from the pressure of temporary causes, between 266, the highest, and 213, the lowest. In 1846, which was the great year of excitement caused by the railway panic, the suicides in London were 266; in 1847 began a slight improvement, and they fell to 256; in 1848 they were 247; in 1849 they were 213; and in 1850 they were 229."—(*History of Civilization,*—pp. 24, 27.)

Truly five years constitute a somewhat narrow basis of observation or illustration for so important a conclusion in respect to the *annual* variations of suicide in the metropolis! But letting this pass, we would mention a remarkably interesting fact or two, connected with the moral statistics of the great city, and which have an immediate bearing upon Mr. Buckle's notions of suicide and crime, although not mentioned by him. From 1701 to 1829 the tendency to suicide in London remained nearly stationary, but the tendency to commit murder rapidly decreased during the same period. In the seventeenth century 4·6 murders occurred in every 10,000 deaths from all causes; in the nineteenth century only 0·5.* These results are obtained from the weekly Bills of Mortality; they are but approximative, but they are quoted and made use of on the authority of Dr. Farr. If then suicide, the product of a general state of society, is to be taken as an index of that state from 1701 to 1829, it would appear that during that period the said state underwent no very manifest change. But murder as well as suicide, indeed crime in general, is said by Mr. Buckle to be "the result, not so much of the vices of the individual offender, as of the state of society into which the individual is thrown."† Consequently the fixed character of the state of

* *London Bills of Mortality.*—Proportion of Deaths from Suicide and Murder in 10,000 Deaths from all causes.

| | Suicide. | Murder. |
|---|---|---|
| 1647 to 1700 | 8.5 | 6.5 |
| 1701 — 1749 | 16.2 | 3.4 |
| 1750 — 1799 | 15.0 | 2.1 |
| 1800 — 1829 | 18.6 | 1.7 |

† Mr. Buckle quotes (*note* p. 37) in support of this conclusion, Quetelet's statement that "Experience demonstrates conclusively this opinion, which might seem society—"the irresistible larger law"—governing suicide, is entirely inconsistent with the progressive and constant change manifested in the state of society governing and necessitating murder in the same period. If then the "state of society" is to be regarded as an equivalent term, as used by Mr. Buckle in reference to both murder and suicide, we are reduced to the dilemma of believing that each crime either comports itself in a fashion of its own towards the general law, and modifies in a constant and regular fashion the action of that law, in which case, what becomes of Mr. Buckle's assertion of its irresistible character? Or that there is a general state of society peculiar to each crime, governed by very different laws, and of which the crime is the product, in which case the phrase may mean anything or nothing (as we have already had occasion to remark), as may be most convenient.

paradoxical at the first sight, *that it is society which prepares crime, and that the criminal is but the instrument which executes it.*" (*Sur l'Homme*, L. iv. c. ii.) But the word "society" is not used by Quetelet and by Mr. Buckle in the same sense. In the sentence preceding that quoted, Quetelet says, that "since the crimes that are annually committed seem to be a necessary result of our social organization, and that the number can diminish only as the causes which lead to them are previously modified, it is for legislators to recognise these causes, and remove them as much as possible." Here social organization is used in the ordinary signification—the moral actions of man being conceived to play a primary part in it; and legislation would (as it does) refer as well to the moral as to the other causes which concur in bringing about a social organization or state of society. But with Mr. Buckle the moral acts of men become entirely subsidiary to the action of "what is called Nature," and they play an ambiguous disturbing effect, not a primary causative effect. Hence his notion of "society" is very different from Quetelet's, and the signification to be attached to that writer's remarks widely varies from that which Mr. Buckle would attach to them, and by the mode in which he quotes them, we regret to say, seems to wish to convey to others. "We must not conclude," writes Quetelet, "from what I have said, that all the actions of man, that all his tendencies, are submitted to fixed laws; and that consequently I suppose his free will to be absolutely annihilated. In order to remove any misconception in this respect, some explanations will be so much the more necessary, since they will throw light upon the question of free-will, one of the most difficult and most interesting questions that occur in the studies which occupy our attention. If, for example, we consider the tendency to crime in man, we mark first that this tendency depends upon his peculiar organization, his education, the circumstances in which he is placed, as well as his free-will, to which I accord willingly the greatest influence in modifying all his propensities . . . . As to the free-will, very far from causing perturbations in the series of phenomena which occur with this admirable regularity, it prevents them, on the contrary, in this sense, that it restrains the limits within which the variations of our different propensities are manifested . . . . Thus, then, free-will, very far from interposing an obstacle to the regular production of social phenomena, favours it on the contrary. A people formed only of sages would exhibit annually the most constant recurrence of the same facts. This will explain that which seems at first a paradox—that is to say, that *social phenomena, influenced by the free-will of man, proceed from year to year with greater regularity than phenomena purely influenced by material and fortuitous causes.*" (*Du Système Social*, pp. 95—97.) Mr. Buckle regards free-will as a metaphysical figment; he conceives this belief to be conclusively supported by statistics; he is evidently not a statistician himself; yet the foregoing are the conclusions of his chief and most highly-lauded statistical authority!

The key to Mr Buckle's specious and inconsequent argument is to be found in the following propositions which precede it:—

"It is evident that, if it can be demonstrated that the bad actions of men vary in *obedience* to the changes in the surrounding society, we shall be obliged to infer that their good actions, which are, as it were, the residue of their bad ones, vary in the same manner; and we shall be forced to the other conclusion, that such variations are the result of large and general causes, which, working upon the aggregate of society [mark the phraseology—causes working upon society, therefore independent of] must produce certain consequences without regard to the volition of those particular men of whom the society is composed.

"Such is the regularity *we expect to find*, if the actions of men are governed by the state of the society in which they occur; *while on the other hand, if we can find no such regularity, we may believe that their actions depend on some capricious and personal principle peculiar to each man, as free will, or the like*" (p. 21):—

That is, Mr. Buckle assumes, *à priori*, that the actions of men, *per se*, are governed by no regular laws, and that they must, of necessity, be manifested in "some capricious" manner; but if it be discovered by observation that the said actions *are* governed by regular laws of recurrence, then it would follow that the *cause* of the said laws must be something apart from, and independent of the individual, consistently with the previous assumption of the eccentricity of his special action. And if, moreover, it be further discovered that there is a certain correspondence between changes in the state of society, and the recurrence of certain actions of men, those actions must be in *obedience* to (not simply concurrent with) the changes in society. Then, murder and suicide being taken, by Mr. Buckle, among other human acts, to illustrate his propositions, and he finding that murder and suicide are apparently governed by regular laws of recurrence under *given* circumstances of society, he at once concludes, in accordance with the propositions, that the regularity is due to the state of society, thus explaining the facts of correspondence by his previous assumption, and asserting the truth of his assumption by the facts which he seeks to explain by it! Mr. Buckle first begs the principle (the key of his entire method of reasoning) which it is necessary to prove, with this principle thus begged he explains the facts he considers, and then he assumes that the facts *thus explained* demonstrate the principle!

And yet it is upon reasoning of this kind that Mr. Buckle seeks to obtain assent to a conclusion which is equivalent to the assertion that suicide is a ghoul-like necessity, against which neither the individual nor the collective efforts of man can avail anything; and wherever Mr. Buckle's reasoning finds acceptance, it may be anticipated that it will lead to an unfortunate indifference to suicide in its social relations. Meriting neither praise nor blame, and uninfluenced by moral restraints, the act must be submitted to as a disagreeable necessity of every-day life, and we must accustom ourselves to it in the best way we can. And how will this be brought about? Shall we rest content to have this revolting creation of a new Frankenstein hunting its victims day by day to death among us in commonplace ghastly guise? Surely not. We shall strive to hide the most horrible features beneath a profusion of conceits; we shall fence in the pathways of the demon with a wealth of fanciful sentiment, and, it may be, we shall end as many others have done (as we shall have in due time to tell), by enshrining an image of him, and worshipping it. In short, the pseudo-philosophy of Mr. Buckle tends towards the same end—the same unhealthy tone of sentiment concerning suicide, which is found to pervade the quotations which we have given from French writers on the subject, and the more intricate workings of which we have still to trace out.

Let us have a care. We have our present artists, who find a charm in suicide; we have an apologist for the act in certainly one of the most facile and attractive historical writers of the day; and the prescriptions of both the law and the gospel in reference to it are, in a great measure, unheeded. This is not a bad starting-point and groundwork in favour of a reactionary movement, sympathetic of suicide; and if we do not take heed, we shall have our young men and maidens looking upon the deed as a matter of feeling, and not of morality. And so, in due time, we should come to hear the legitimacy of suicide babbled of at our fire-sides and in our workshops, while sympathy would find an outlet in song. Would you have an example of the song? Read—

I.

Up, up, my page! and saddle quick,
And mount my fleetest steed,
And over field, and over fell,
To Duncan's castle speed.

Lurk in the stable till thou spy
Some horse-boy of the train,
Then ask him, which the bride may be
Of Duncan's daughters twain?

And should he say, "The olive maid,"
Ride back without delay;
But should he say, "The fair-haired girl,"
Then linger by the way.

Then hie thee to the rope-yard, boy,
And purchase me a cord:
Ride slowly home, and give it me,
But do not speak a word.

II.

The suicide lies at the cross-roads,
Interr'd at the midnight hour;
And there a blue floweret blossoms—
The poor sinner's flower.

I stood at the cross-roads sighing;
'Twas hard on the midnight hour;
There waved in the moonlight slowly
The poor sinner's flower.*

"Give me an ounce of civet, good apothecary, to sweeten my imagination."

We have not reached the core of our subject, and yet we are at the end of our space. We hope, however, at another time to pursue our theme.

* Heinrich Heine's Book of Songs. Translated by John L. Willis. London: 1856.

